# ROS-Dependent Antiproliferative Effect of Brassinin Derivative Homobrassinin in Human Colorectal Cancer Caco2 Cells

**DOI:** 10.3390/molecules190810877

**Published:** 2014-07-25

**Authors:** Martin Kello, David Drutovic, Martina Chripkova, Martina Pilatova, Mariana Budovska, Lucia Kulikova, Peter Urdzik, Jan Mojzis

**Affiliations:** 1 Department of Pharmacology, Faculty of Medicine, Pavol Jozef Safarik University, 040 11 Kosice, Slovak Republic; 2 Department of Organic Chemistry, Institute of Chemical Sciences, Faculty of Science, Pavol Jozef Safarik University, 040 80 Kosice, Slovak Republic; 3 Department of Experimental Medicine, Faculty of Medicine, Pavol Jozef Safarik University, 040 11 Kosice, Slovak Republic; 4 Department of Gynaecology and Obstetrics, Faculty of Medicine, Pavol Jozef Safarik University, 040 11 Kosice, Slovak Republic; 5 L. Pasteur University Hospital, 040 11 Kosice, Slovak Republic

**Keywords:** indole phytoalexins, homobrassinin, antiproliferative, apoptosis, ROS

## Abstract

This study was designed to examine the *in vitro* antiproliferative effect of brassinin and its derivatives on human cancer cell lines. Among seven tested compounds, homobrassinin (**K1**; N-[2-(indol-3-yl)ethyl]-S-methyldithiocarbamate) exhibited the most potent activity with IC_50_ = 8.0 μM in human colorectal Caco2 cells and was selected for further studies. The flow cytometric analysis revealed a **K1**-induced increase in the G_2_/M phase associated with dysregulation of α-tubulin, α_1_-tubulin and β_5_-tubulin expression. These findings suggest that the inhibitory effect of **K1** can be mediated via inhibition of microtubule formation. Furthermore, simultaneously with G_2_/M arrest, **K1** also increased population of cells with sub-G_1_ DNA content which is considered to be a marker of apoptotic cell death. Apoptosis was also confirmed by annexin V/PI double staining, DNA fragmentation assay and chromatin condensation assay. The apoptosis was associated with the loss of mitochondrial membrane potential (MMP), caspase-3 activation as well as intracellular reactive oxygen species (ROS) production. Moreover, the antioxidant Trolox blocked ROS production, changes in MMP and decreased **K1** cytotoxicity, which confirmed the important role of ROS in cell apoptosis. Taken together, our data demonstrate that **K1** induces ROS-dependent apoptosis in Caco2 cells and provide the rationale for further *in vivo* anticancer investigation.

## 1. Introduction

A plethora of epidemiological and animal studies show that consumption of cruciferous vegetables may lower the risk for variety of cancers [1,2,3,4]. It is suggested that the cancer-protective effects of cruciferous vegetables can be associated with the presence of glucosinolates, which are cleaved to biologically active compounds, such as indoles and isothiocyanates [5].

Another group of cruciferous-derived phytochemicals, the indole phytoalexins, have attracted scientists’ interest because of their ability to modulate processes involved in oncogenic transformation, such as alterations of cell cycle control, apoptosis evasion and inhibition of different signalling pathways [6,7,8].

Generally, phytoalexins are low molecular weight secondary metabolites biosynthesized *de novo* by plants in response to stress caused by biotic or abiotic factors [9,10]. Although phytoalexins are part of general defense mechanisms used to ward off plant invaders, their chemical diversity suggest substantially broader biological activities. In addition to their antimicrobial activity, some phytoalexins also possess antiinflammatory [11], antioxidant [12], antiproliferative [13,14], as well as anticancer [15,16] properties.

Indole phytoalexins are structurally unique, sulfur-containing natural products isolated from plants of the family Cruciferae (syn. Brassicaceae). Besides their antimicrobial properties, several indole phytoalexins also exhibit antiproliferative/anticancer activity [17,18,19,20,21]. 

Brassinin ([3-(S-methyldithiocarbamoyl) aminomethyl indole]), first isolated from Chinese cabbage [22], is an indole phytoalexin with demonstrated antiproliferative/anticancer activity. Mehta and co-workers [23] documented dose-dependent inhibition of 7,12-dimethylbenz[a]anthracene (DMBA)-induced preneoplastic lesion formation by brassinin and cyclobrassinin in a mouse mammary gland organ culture model. Later, Csomós *et al.* [24] showed antiproliferative effects of brassinin, isobrassinin and isobrassinin derivatives in different cancer cell types. Recently, Izutani *et al.* [6] described the ability of brassinin to inhibit cell growth in human colon cancer cells by arresting the cell cycle at the G_1_ phase via increased expression of p21 and p27. In the last decade we have also documented the antiproliferative effects of brassinin or its derivatives in different cancer cells [25,26,27,28,29,30].

Although the precise mechanism(s) of the antiproliferative activity of brassinin and its derivatives still remain unknown, inhibition of indoleamine 2,3-dioxygenase and inhibition of PI3K/Akt/mTOR signalling pathways may interfere with cancer cell survival and proliferation [7,31]. However, so far there is no published information about the antiproliferative molecular mechanisms of homobrassinin on cancer cells. 

It is well known that oxidative stress may play role in the cytotoxicity of different natural compounds [32,33]. Recently, it was documented that the antiproliferative effect of some indole phytoalexins may be associated with ROS production [34,35] or glutathione depletion [19,30], which may lead to imbalance between antioxidant and prooxidant factors. This prompted us to explore the role of ROS in the antiproliferative effects of brassinin and its derivatives. Our results demonstrate that homobrassinin (**K1**) is the most active in inhibiting the growth of Caco2 cells among the compounds studied. Effect of **K1** is associated with ROS production leading to mitochondrial dysfunction, caspase 3 activation and apoptosis induction. The role of ROS in **K1**-induced cell death was analysed by intracellular ROS generation and ROS scavenger experiments. These findings generate a rationale for *in vivo* efficacy studies with this compound in preclinical cancer models.

## 2. Results and Discussion

### 2.1. Effect of Brassinin and Its Derivatives on Cell Proliferation

**Figure 1:**
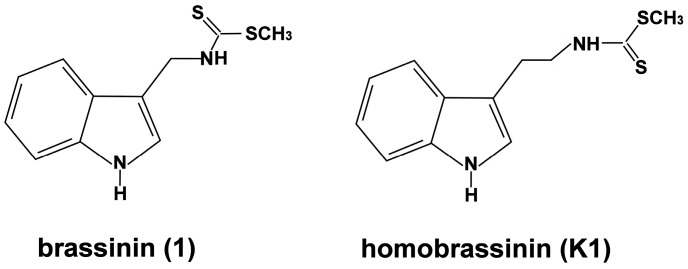
Chemical structure of brassinin (**1**) and homobrassinin (**K1**).

The antiproliferative effect of indole phytoalexins was evaluated on eight human cancer cell lines using the MTT assay. Survival of cancer cells exposed to the studied indole phytoalexins is shown in Table 1. Our data showed that brassinin (**1**, Figure 1) possesses relatively weak antiproliferative effect with IC_50_ > 100 μM in all cancer cell lines used. Similar results were obtained also with compounds **K10** and **47**. On the other hand, homobrassinin (**K1**, Figure 1) displayed the highest antiproliferative activity with IC_50_ from 8.0 to 35.0 μM with the greatest antiproliferative activity in Caco2 cells. Other indole phytoalexins (**K49**, **K124** and **K170**) were less potent.

**Table 1:** The IC_50_ (μM) of tested compounds in different cell lines after 72 h incubation. Results are presented as a mean ± SD of three independent experimental determinations performed in triplicate. The tested compounds: Brassinin (**1**), Homobrassinin (**K1**), *N*-{[1-(*tert*-Butoxycarbonyl)indol-3-yl]methyl}-*N*'-methyl-N'-phenylthiourea (**K10**), *N*-{[(1-*tert*-Butoxycarbonyl)indol-3-yl]metyl}-*N*'-(4-methoxyphenyl)thiourea (**K124**), 1-(β-d-glucopyranosyl)brassinin (**47**), 1-[(1*R*,2*S*,5*R*)-Menthoxycarbonyl]brassinin (**K49**); 1-[(1*R*,2*S*,5*R*)-8-Phenylmenthoxycarbonyl]brassinin (**K170**).

Compound	Cancer Cell Lines							
	Jurkat	Caco2	HepG2	HCT-116	A549	HeLa	MCF-7	MDA-MB-231
**1**	>100	>100	>100	>100	>100	>100	>100	>100
**K1**	28.2 ± 1.2	8.0 *±* 0.6	21.3 *±* 2.3	27.3 *±* 1.8	33.4 *±* 0.8	26.1 *±* 2.4	35.0 *±* 2.1	22.8 *±* 1.4
**K10**	>100	>100	>100	>100	>100	>100	>100	>100
**K124**	30.0 *±* 0.5	>100	>100	30.7 *±* 2.9	>100	34.0 *±* 0.7	92.0 *±* 2.7	>100
**K49**	39.6 *±* 1.8	89.4 *±* 1.3	37.4 *±* 3.7	76.0 *±* 2.5	22.8 *±* 0.5	45.6 *±* 1.4	62.0 *±* 3.4	>100
**K170**	44.3 *±* 3.2	>100	>100	53.7 *±* 2.1	96.5 *±* 4.1	>100	>100	>100
**47**	>100	>100	>100	>100	>100	>100	>100	>100

Because only **K1** displayed interesting antiproliferative activity, it was selected for further mechanistic studies in Caco2 cells. The antiproliferative activity of compound **K1** was also compared with its activity against non-cancer cells, human umbilical vein endothelial cells (HUVEC). Our results shown that compound **K1** was more active against cancer cells than non-cancer cells (IC_50_ = 8.0 *vs.* IC_50_ = 46.2 μM).

**Figure 2:**
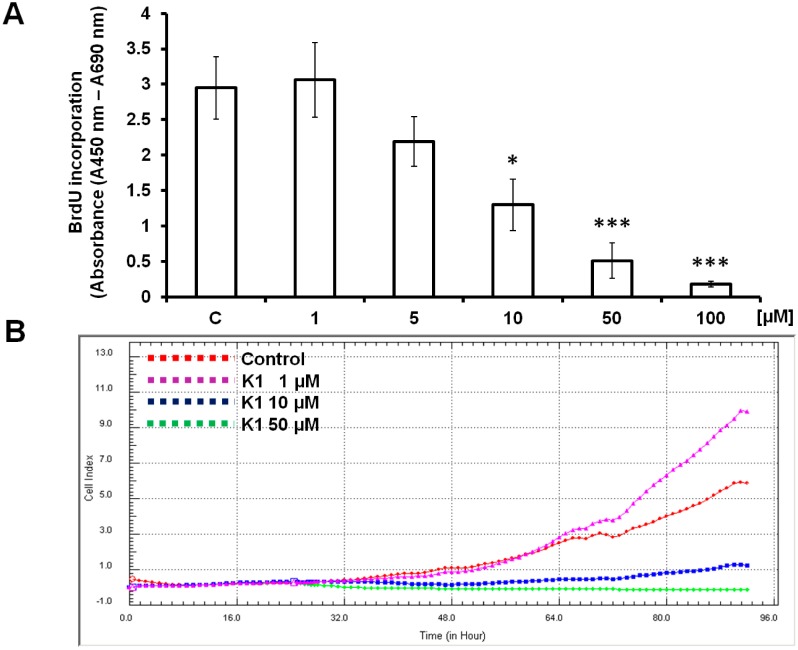
Antiproliferative effect of compound **K1** on Caco2 cells. (**A**) Effect of 72 h **K1** treatment on proliferation of Caco2 cells, as measured by the BrdU ELISA proliferation assay, *** *p* < 0.001, * *p* < 0.05. Data were obtained from three independent replicate experiments with at least three wells per treatment group in each individual replicate. (**B**) Real-time monitoring of Caco2 cell proliferation after incubation with **K1** using the xCELLigence system. Appropriate concentrations of **K1** were added 24 h after seeding.Representative picture from three independent experiments is shown.

To confirm the potential antiproliferative effect of **K1**, the BrdU proliferation assay was used. The magnitude of the absorbance for the developed colour is proportional to the quantity of BrdU incorporated into cells, which is a direct indication of cell proliferation. As shown in Figure 2A, **K1** at concentrations of 100, 50 and 10 μM significantly decreased BrdU incorporation compared with the control (approximately 94%, 83% and 56% respectively) (*p* < 0.001; *p* < 0.05). Antiproliferative effect was not observed at the concentration of 1 μM.

The antiproliferative effect of compound **K1** on Caco2 cells was also evaluated by xCELLigence system. As shown in Figure 2B the cell index of Caco2 cells decreased significantly after treatment with **K1** in a concentration-dependent manner. However, the cells treated with 1 μM of **K1** exhibited a similar proliferation rate as control for most of the duration of the experiment, which confirm results obtained by BrdU proliferation assay.

### 2.2. K1 Blocks Cell Cycle at G_2_/M Transition

To evaluate the effect of **K1** treatment on Caco2 cell cycle progression, we performed flow cytometric analysis on cells treated with 10 μM **K1** for 24, 48 and 72 h as described in the Experimental Section. Results obtained are summarized in Table 2. The significant enrichment in G_2_/M cell populations was observed after 24 h (70.48%) of **K1** treatment, as compared to untreated cells (36.64%) (*p* < 0.001). As a consequence, a significant reduction of the G_0_/G_1_ phase cell population confirms the cell cycle arrest in G_2_/M as an effect of colorectal cells exposure to **K1**. After 48 and 72 h incubation G_2_/M arrest still persisted with addition of increased sub-G_1_ population of cells. These results strongly suggest that **K1**-induced cell cycle arrest may represent one of the mechanisms by which this indole phytoalexin inhibits colorectal cancer cells growth.

**Table 2:** The distribution of cell cycle in Caco2 cells treated with **K1**. Cells were treated with **K1** derivate (c = 10 µM) for 24, 48 and 72 h. The distribution of cell cycle was assessed by flow cytometry. Each value is the mean ± SD of three independent experiments. The significant differences between control and **K1**-treated cells were signed as *p* < 0.05 (*), *p* < 0.01 (**), *p* < 0.001 (***).

Treatment	Time (h)	Sub-G_1_	G_0_/G_1_	S	G_2_/M
Control		0.29 ± 0.15	40.81 ± 3.29	18.25 ± 3.90	36.64 ± 2.29
K1	24	1.49 ± 0.37	21.34 ± 3.13 **	6.69 ± 1.56 *	70.48 ± 2.28 ***
K1	48	3.36 ± 1.67 *	30.89 ± 2.81 *	9.58 ± 2.43	56.20 ± 2.58 **
K1	72	5.58 ± 1.16 *	30.17 ± 1.65 *	11.47 ± 1.65	52.79 ± 2.77 **

### 2.3. K1 Induces Apoptotic Cell Death

**Figure 3:**
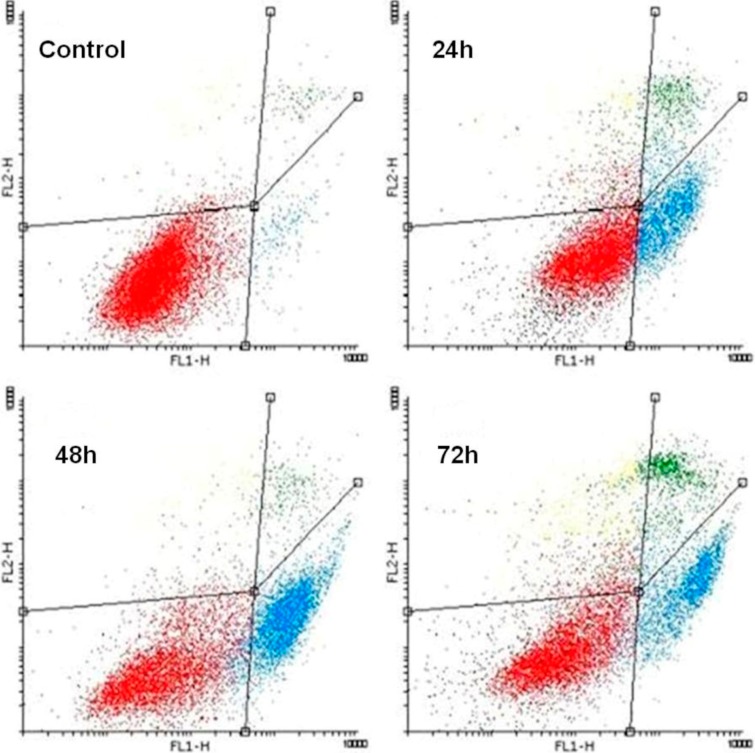
Induction of apoptosis after **K1** treatment measured by Annexin V/PI staining. Caco2 cells were treated with **K1** for 24, 48 and 72 h, stained with fluoresceinated Annexin V and PI, and analysed by flow cytometry. One representative experiment out of three is shown. The percentage of events in the nonapoptotic (lower left, An^−^/PI^−^), early apoptotic (lower right, An^+^/PI^−^), and late apoptotic/necrotic (upper left + right, An^−^/PI^+ ^ + An^+^/PI^+^) quadrants is indicated in Table 3.

The increase of cell having sub-G_1_ DNA content is considered to be a marker of apoptotic cell death. To confirm whether **K1** could induce apoptosis on colorectal cell lines, we performed Annexin V/PI staining, which detects an early stage of apoptosis and combined staining with PI, which detects a late stage of apoptosis or necrosis. The results showed that only a small percentage of untreated Caco2 cells (3.17%) bound with annexin V. In contrast, the percentage of annexin V binding Caco2 cells significantly increased in a time-dependent manner after 24, 48 and 72 h of treatment with 10 μM **K1** (18.54% to 37.07%, *p* < 0.01; *p* < 0.001 ). Simultaneously, percentage of annexin V/PI positive cells increase from 2.05% (untreated cells) to 20.85% (**K1** treated cells after 72 h of incubation). These experimental results demonstrate that **K1** induced apoptosis of Caco2 cells (Table 3; Figure 3).

**Figure 4:**
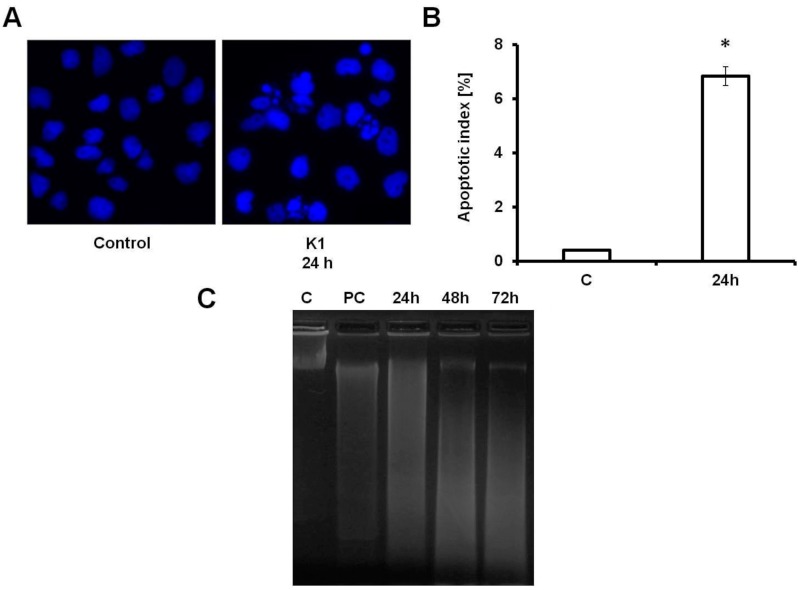
Apoptotic effect of **K1** on Caco2 cells. (**A**) Morphological changes of Caco2 cells treated with 10 μM of **K1** for 24 h. Cells were cultured in chamber slides and stained with DAPI to show typical apoptotic morphology. Magnification 400×. (**B**) Apoptotic index of Caco2 cells treated with 10 μM of **K1** for 24 h. The results (mean ± SD) of three independent experiments are shown as the apoptotic index evaluated as a percentage of cells with fragmented nuclei from a total number of minimum 300 cells, * *p* < 0.05. (**C**) DNA fragmentation of Caco2 cells after incubation with 10 μM of **K1** for 24, 48 and 72 h, C-control (untreated cells), PC-positive control (cells treated with 10 μM of cisplatin). A representative picture from three independent experiments is shown.

Chromatin condensation is one of the most important markers for apoptotic cells. The nuclear morphological changes of Caco2 cells were analysed using DAPI staining. In control groups, cells appeared to be round and manifested homogeneous nuclei. In cells treated with **K1** at concentration 10 μM, cells displayed condensed nuclei. A significant increase of cells with typical apoptotic morphology was observed (Figure 4A), reaching 6.83% after 24 h of treatment, as evaluated by determination of apoptotic index (Figure 4B).

**Table 3:** Induction of apoptosis after **K1** treatment measured by Annexin V/PI staining Caco2 cells were treated with **K1** for 24, 48 and 72 h, stained with fluoresceinated Annexin V and PI, and analysed by flow cytometry. The percentage of events in the nonapoptotic (lower left, An^−^/PI^−^), early apoptotic (lower right, An^+^/PI^−^), and late apoptotic/necrotic (upper left + right, An^−^/PI^+^ + An^+^/PI^+^) quadrants (Figure 3) is indicated. The significant differences between control and **K1**-treated cells were signed as *p* < 0.05 (*), *p* < 0.01 (**), *p* < 0.001 (***).

Treatment	Time (h)	An^−^/PI^−^	An^+^/PI^−^	An^+^/PI^+^
Control		94.07 ± 1.32	3.17 ± 0.60	2.05 ± 0.50
K1	24	67.81 ± 1.18 **	18.54 ± 1.63 **	13.75 ± 2.43 *
K1	48	49.92 ± 3.20 ***	38.55 ± 2.47 ***	11.81 ± 2.24 *
K1	72	42.36 ± 3.65 ***	37.07 ± 2.66 ***	20.85 ± 3.69 **

Analysis of DNA fragmentation by agarose gel electrophoresis is one of the most widely used biochemical markers for cell death. Fragmentation of DNA was observed in Caco2 cells after 24 h incubation with 10 μM of compound **K1** compared to control (untreated cells). This effect also persisted after 48 and 72 h of incubation (Figure 4C).

### 2.4. Effect of K1 on ROS Formation

**Figure 5:**
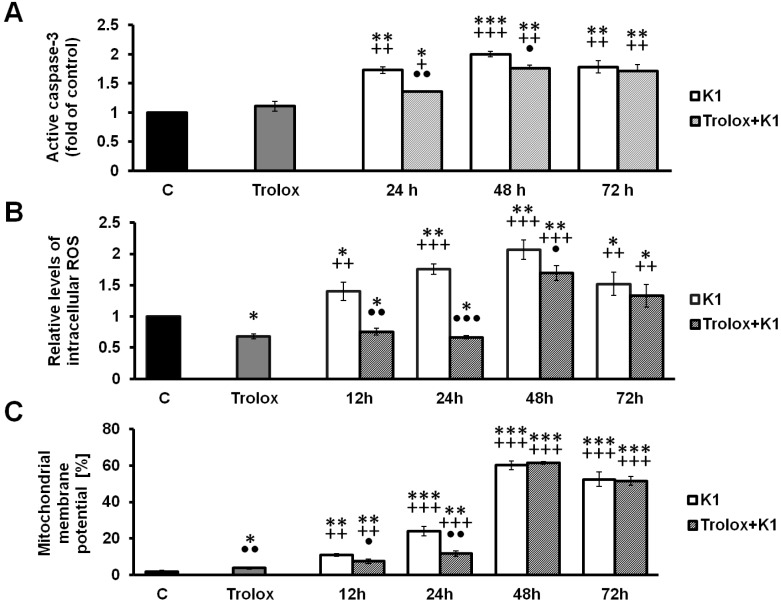
Effect of **K1** and Trolox on caspase-3, ROS accumulation and MMP. (**A**) Effect of **K1** and Trolox treatment on caspase-3 activation. Caspase-3 activation was measured 24, 48 and 72 h after treatment by quantifying the fluorescence intensity. (**B**) Measurement of ROS production in Caco2 cells treated with **K1**, Trolox or mutual combinations. Cytosolic ROS were measured 12, 24, 48 and 72 h after treatment by quantifying the fluorescence intensity of activate DHR-123. The results (mean ± SD) of three independent experiments are shown as multiples of the control group fluorescence. (**C**) MMP changes in Caco2 cells treated with **K1**, Trolox or mutual combinations were analysed 12, 24, 48 and 72 h after treatment.

**Figure 6:**
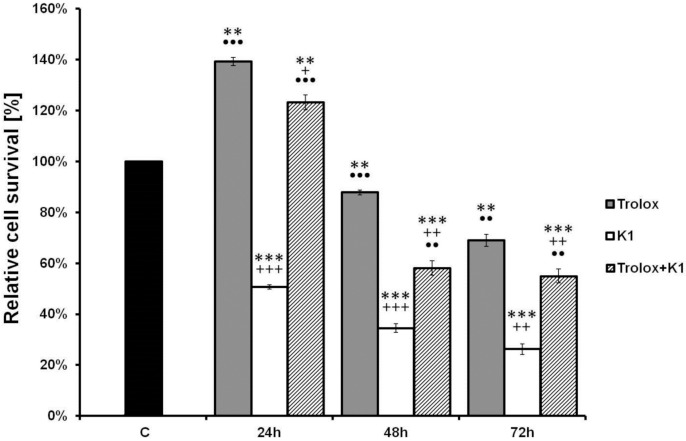
Relative survival of Caco2 cells treated with **K1**, Trolox or mutual combinations as evaluated by MTT assay. Cells were incubated with tested compounds for 24, 48 and 72 h.

It has been reported that increased liberation of ROS can activate a cascade of events leading to apoptosis [36]. Therefore, we decided to detect whether K1 was able to trigger ROS generation by measuring the rhodamine 123 fluorescence intensity. The results showed that treatment of the cells with **K1** significantly increased ROS generation in a time-dependent manner. After 12 h incubation with **K1** the significant ROS accumulation was observed. Culminated accumulation was marked after 48 h treatment (Figure 5B). On the other hand, no increase in ROS production was observed after 1, 3 and 6 h of incubation (data not shown). In order to show that generation of ROS is a key step in the **K1**-induced apoptotic pathway, Caco2 cells were pretreated with Trolox, a water-soluble analogue of the free radical scavenger α-tocopherol. Trolox significantly decreased ROS accumulation in time dependent manner. These effects were associated also with recovered cell viability (Figure 6).

Results are expressed as mean values ± SD of three independent experiments. * *p* < 0.05, ** *p* < 0.01, *** *p* < 0.001 *vs.* untreated control; ^+^ *p* < 0.05, ^++^ *p* < 0.01, ^+++^ *p* < 0.001 *vs.* Trolox; ^●^ *p* < 0.05, ^●●^ *p* < 0.01, ^●●●^ *p* < 0.001 *vs.* **K1**.

Collectively, these findings suggest that an increase in ROS generation may take part in the **K1**-induced apoptosis in Caco2 cells.

### 2.5. K1-Induced Mitochondrial Dysfunction

A decrease in MMP is one of the earliest events in apoptosis. Mitochondrial membrane integrity was evaluated using the cationic dye TMRE, a highly specific probe for detecting changes in mitochondrial ΔΨ_m_. In our experiments, changes in MMP were detected 12, 24, 48 and 72 h after **K1** treatment. Effect of **K1** treatment on Caco2 cells led to intensive and considerable destruction of mitochondrial function demonstrated with significant changes in the percentage of cells with dissipated MMP in time-dependent manner. On the other hand, co-treatment with Trolox significantly diminished effect of **K1** on MMP changes and prevented mitochondrial dysfunction (Figure 5C).

Data is presented as a mean ± SD of three independent experimental determinations performed in triplicate. ** *p* < 0.01, *** *p* < 0.001 *vs.* untreated control; ^+^ *p* < 0.05, ^++^ *p* < 0.01, ^+++^ *p* < 0.001 *vs.* Trolox; ^●●^ *p* < 0.01, ^●●●^ *p* < 0.001 *vs.* **K1**.

### 2.6. Activation of Caspase-3 by K1

Analysis of caspase-3 activation (Figure 5A) clearly demonstrated caspase-dependent form of cell death in Caco2 cells line. The more than 2-fold higher increase of caspase-3 activation after 48 h treatment confirmed this suggestion. On the other hand, co-treatment with Trolox significantly decreased the effect of **K1** on caspase-3 activation. Significant caspase-3 activation correlated well with apoptosis occurrence frequency, ROS and loss of MMP leading to cell death after **K1** treatment. These results clearly indicate that caspase-3 activation plays an important role in Caco2 cell apoptosis induced by **K1**.

### 2.7. Effect of K1 on Gene Expression of Tubulins

The cell cycle analysis showed that **K1** induces the G_2_/M arrest. Based on this result, the alteration in selected gene expression was analysed after 24, 48 and 72 h incubation by real-time PCR. We found significant downregulation of β-tubulin expression and significant upregulation of α-tubulin expression after 24 h treatment with compound **K1** at concentration 10 μM. Similar results were also obtained after 72 h of incubation. After 48 h incubation with the tested compound an opposite but non-significant effect was noted (Table 4). 

**Table 4:** Fold changes of specific genes after 24, 48 and 72 h treatment with **K1** (c = 10 μM). β-actin gene was used as a housekeeping gene to normalize each sample.

Genes	Normalized Ratio		
	24 h	48 h	72 h
α-tubulin	5.59	1.68	3.35
α_1_-tubulin	1.89	0.65	1.47
β_5_-tubulin	0.29	0.99	0.48

These results suggest that **K1** may affect microtubule and microtubule assembly. The results of real-time PCR analysis for selected genes are in direct correlation with the data obtained in cell cycle analysis.

### 2.8. Effect of K1 on Cytoskeletal Tubulins Assembly

**Figure 7:**
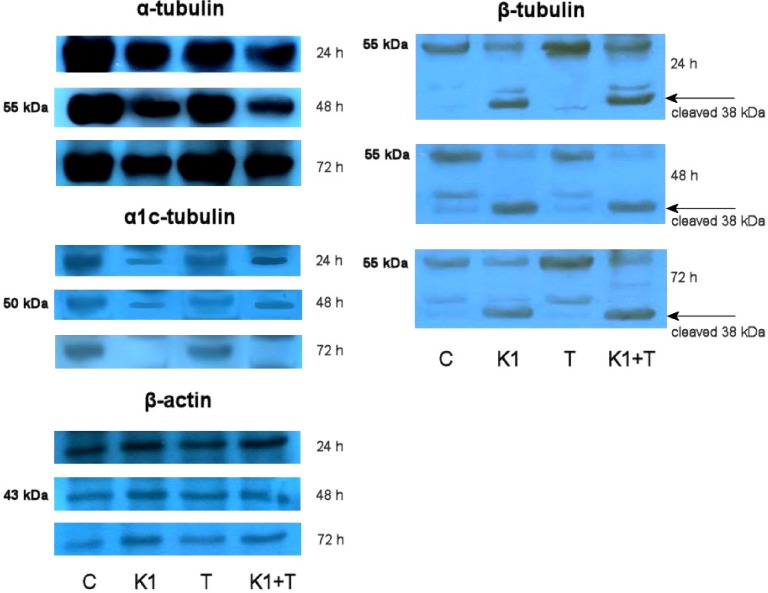
Western blot detection of α and β tubulins in Caco2 cells treated with **K1**, Trolox or mutual combinations. Cells were incubated with tested compounds for 24, 48 and 72 h. Representative picture from three independent experiments is shown. β-Actin served as a loading control.

To determine whether the tubulins are involved in **K1**-mediated apoptosis of Caco2 cells, western blotting was used to detect cellular changes of α, α1c and β tubulins from whole cell lysates. All α, α1c and β tubulin levels showed a time-dependent decrease after treatment with **K1** (Figure 7). 

The β tubulin analysis also confirmed degradation and cleaving of presented protein to lower mass form. The results from both 48 and 72 h treatments indicated that tubulin levels decreased rapidly, and the effects proceeded for a long time. The correlation between changes of tubulins level and cell cycle distribution suggests that tubulin degradation may be involved in cell cycle arrest and apoptosis after **K1** treatment. To explain the possible involvement of oxidative stress in the tubulin degradation, disaggregation or deregulation we used Trolox as an antioxidant to prevent an oxidative burst after **K1** treatment. Data showed that Trolox alone has minimal or no effect on tubulin assembly. In combination, Trolox with **K1** treatment is not able to diminish the effect of **K1** on tubulin levels. Only short time treatment (24 h) with **K1** and Trolox at the β tubulins level pointed to a minor Trolox effect compared with **K1** treatment.

### 2.9. Discussion

Cancer, characterized by unregulated proliferation of cells, is the second major cause of death after cardiovascular disease [37]. Despite enormous progress in the understanding of carcinogenesis, the discovery of anticancer drugs remains a highly challenging endeavor. Natural products provide one of the most important sources of promising leads for the development of novel chemotherapeutics.

In the present study, the antiproliferative effects of brassinin and some of its synthetic derivatives on cancer cells were investigated. Our results revealed that the natural or synthesized compounds exhibited different levels of antiproliferative activity in cancer cells. Compound **K1**, in particular, showed much higher levels of bioactivity than naturally occurring brassinin, with IC_50_ = 8.0 μM in human colorectal adenocarcinoma Caco2 cells. Interestingly, **K1** at lower concentration stimulated cancer cell proliferation. Similar biphasic, dose-dependent effect of natural compounds or their chemical analogues have been documented only in a limited number of studies. Polyphenols such as apigenin, quercetin, genistein or red wine polyphenols at low concentrations stimulated cell growth. On the contrary, at the higher concentrations decrease in cell proliferation was observed [38,39,40,41].

To our knowledge, this is the first report of an investigation into the effect of **K1** on proliferation in cancer cells. We demonstrated that **K1**-induced antiproliferative effect in human colorectal Caco2 cells was related to cell cycle arrest and increase of cells with sub-G_1_ DNA content and induction of apoptosis, as confirmed by annexin V/PI staining, measurement of chromatin condensation, DNA fragmentation and caspase 3 activation. Our data also indicated that **K1** induced apoptosis of Caco2 cells through generation of ROS and mitochondrial dysfunction, suggesting that ROS act as upstream signalling molecules for initiation of cell death.

Flow cytometric analysis showed that the **K1**-induced decrease in Caco2 cell viability is associated with cell cycle arrest in the G_2_/M phase. This result prompted us to analyse expression of selected genes involved in microtubules formation. Microtubules are built up of a heterodimer of α- and β-tubulin. These constantly growing and shortening dynamic structures are involved in many important cellular processes particularly associated with cell division [42]. Their importance in mitotic spindle formation and chromosome movement during cell division which is uncontrolled in cancer cells makes tubulin an important target for cancer therapy. The effect of the most successful antineoplastic drugs called as tubulin inhibitors, such as the taxanes and the vinca alkaloids, interfere directly with the tubulin system [43]. The tested compound **K1** increased expression of α-tubulin and decreased expression of β-tubulin. Dysregulated expression of tubulins can lead to insufficiency of α/βhetero-dimer production, material necessary for mitotic spindle formation, resulting in the G_2_/M cell cycle arrest, eventually resulting in apoptotic cell death [44]. Furthermore, **K1** treatment led to degradation or cleavage of corresponding proteins in time dependent manner (Figure 7). Presented destruction of cytoskeletal tubulin assembly also correlates with the observed apoptosis progression.

In addition to G_2_/M cell cycle arrest, the increase of cells with sub-G_1_ DNA content was observed. Compared with the control, cells with sub-G_1_ DNA content were increased 5.1-, 11.5- and 19.2-fold after 24, 48 and 72 h of treatment, respectively. The appearance of the sub-G_1_ fraction and conventional morphological signs of apoptosis provided support for the idea that **K1** induced apoptosis in Caco2 cells (Figure 4). Pro-apoptotic effect of **K1** was further confirmed by annexin V/PI staining. The percentage of apoptotic cells increased in a time-dependent manner (Figure 3). These findings also supported caspase 3 activation observed after **K1** treatment that could be one of crucial step to execution of apoptotic features.

Mitochondria are crucial for energy production, intermediary metabolism, and calcium homeostasis [45]. On the other hand, mitochondrial dysfunction has been recognized as one of the key events occurring at the initial stages of apoptosis [46]. Several experimental studies reported a fall in the MMP during apoptosis induced by various compounds with anticancer properties [47,48,49]. Therefore, MMP changes after **K1** treatment in Caco2 cells were measured. 

In our study, the decrease of MMP was detected 12 h after **K1** treatment, suggesting **K1** induced cell apoptosis in Caco2 cells might be mitochondria dependent (Figure 5). Mitochondria are also the major sites for ROS production. It is well known that excessive generation of ROS may result in cell death [50,51]. Our results showed **K1** treatment significantly stimulated ROS generation in Caco2 cells (Figure 5). To confirm role of ROS in **K1**-induced apoptosis and cell death, Caco2 cells were pretreated with the antioxidant Trolox. Compared to **K1** treatment only, Trolox pretreatment caused a reduction in ROS levels and prevented loss of MMP as well as significantly rescued **K1**-induced Caco2 cytotoxicity (Figure 6). We have also demonstrated that peroxyl radical scavenging does not affect **K1**-mediated tubulin degradation, but we cannot disprove the involvement of other free radical forms in the shortened time of treatment shown in Figure 7.

## 3. Experimental Section

### 3.1. Test Compounds

Brassinin (**1**), homobrassinin (**K1**), N-{[1-(*tert*-butoxycarbonyl)indol-3-yl]methyl}-N'-methyl-N'-phenylthiourea (**K10**), N-{[(1-*tert*-butoxycarbonyl)indol-3-yl]methyl}-N'-(4-methoxyphenyl)thiourea (**K124**), 1-(β-d-glucopyranosyl)brassinin (**47**), 1-[(1R,2S,5R)-menthoxycarbonyl]brassinin (**K49**); 1-[(1R,2S,5R)-8-phenylmenthoxycarbonyl]brassinin (**K170**). The synthesis of tested compounds was described in the previous studies: **1**, **K1** [52]; **K10**, **K124** [28]; **47** [53]; **49**, **K170** [54].

### 3.2. Cell Culture

The human cancer cell lines HCT116 (human colorectal carcinoma), HepG2 (human hepatocellular carcinoma), HeLa (human cervical adenocarcinoma), Jurkat (human leukemic T cell lymphoma) were cultured in RPMI 1640 medium (PAA Laboratories, Pasching, Austria) and Caco2 (human colorectal adenocarcinoma), A549 (human alveolar adenocarcinoma), MCF-7 (human Caucasian breast adenocarcinoma) and MDA-MB-231 (human mammary gland adenocarcinoma) were maintained in growth medium consisting of high glucose Dulbecco’s Modified Eagle Medium (Invitrogen, Carlsbad, CA, USA). Both media were supplemented with a 10% fetal bovine serum (FBS), penicillin (100 IU/mL) and streptomycin (100 μg/mL) (all from Invitrogen). The cells (obtained from the American Tissue Culture Collection, ATCC, Rockville, MD, USA) were maintained under standard tissue culture conditions of 37 °C, 95% air/5% CO_2_. Cell viability, estimated by trypan blue exclusion, was greater than 95% before each experiment. Human umbilical vein endothelial cells (HUVECs) were isolated and cultured as previously described by Ivanova *et al.* [55].

### 3.3. Growth Inhibition Assay

The antiproliferative effects of compounds were determined using colorimetric microculture assay with the MTT end-point [56]. Briefly, 3 × 10^3^ cells were plated per well in 96-well polystyrene microplates (Sarstedt AG & Co, Nümbrecht, Germany) in the culture medium containing tested chemicals at final concentrations of 10^−4^–10^−6^ mol/L or Trolox (6-hydroxy-2,5,7,8-tetramethylchroman-2-carboxylic acid; Fluka, Buchs, Schwitzerland) at final concentrations 300 μM, or at mutual combinations. After 72 h of incubation (for Trolox and mutual combinations also after 24 and 48 h), 10 μL of MTT (5 mg/mL) (Sigma-Aldrich Corporation, St. Louis, MO, USA) were added in each well. After an additional 4 h, during which insoluble formazan was produced, 100 μL of 10% sodium dodecyl sulfate were added in each well and another 12 h were allowed for the dissolution of formazan. The absorbance was measured at 540 nm using the automated uQuant ™ Universal Microplate Spectrophotometer (Biotek, Winooski, VT, USA). The blank-corrected absorbance of the control wells was taken as 100% and the results were expressed as a percentage of the control. All experiments were performed in triplicate. Due to spontaneous apoptosis, HUVEC cells were incubated only 48 h.

### 3.4. 5-Bromo-2'-Deoxyuridine (BrdU) Cell Proliferation Assay

Cell proliferation activity was directly monitored by quantification of BrdU incorporated into the genomic DNA during cell growth. DNA synthesis was assessed using colorimetric cell proliferation ELISA assay (Roche Diagnostics GmbH, Mannheim, Germany) following the vendor’s protocol. Briefly, 2 × 10^3^ cells/well in 80 µL medium were plated in a 96-well polystyrene microplates (Sarstedt AG & Co, Nümbrecht, Germany). Twenty-four hours after cell seeding different concentrations (10^−4^–10^−6^ mol/L) of the compound were added. After 48 h of treatment, cells were incubated with BrdU labeling solution (10 µM final concentration) for another 24 h at 37 °C followed by fixation and incubation with anti-BrdU peroxidase conjugate for an additional 1.5 h at room temperature. Finally, after substrate reaction, the stop solution was added (25 µL 1 M H_2_SO_4_) and colour intensity was measured with multi-well microplate ELISA reader at 450 nm (reference wavelength: 690 nm).

### 3.5. xCELLigence Cell Analysis System

The xCELLigence system is a unique, impedance-based system for cell-based assays, allowing for label-free and real-time monitoring of cellular processes such as cell growth, proliferation, cytotoxicity, adhesion, morphological dynamics and modulation of barrier function. It measures impedance changes in a meshwork of interdigitated gold microelectrodes located at the well bottom (E-plate) or at the bottom side of a microporous membrane (CIM16-plate). These changes are caused by the gradual increase of electrode surface occupation by (proliferated/ migrated/invaded) cells during the course of time and thus can provide an index of cell viability, migration and invasion. This method of quantification is directly proportional to cellular morphology, spreading, ruffling and adhesion quality as well as cell number [57,58].

The xCELLigence RTCA system was initialized, as per manufacturer’s instructions, prior to commencement of the experiment by filling all 16 wells of the E-plate (ACEA Biosciences, San Diego, CA, USA) with the growth medium (100 μL) and equilibrated at room temperature for 30 min. The plate was placed into the single plate (SP) station cradle (housed in a humidified incubator at 37 °C with a 5% CO_2_ atmosphere) to establish a background reading. Then, Caco2 cells were seeded in E-plates at a density of 2 × 10^3^ cells per well. After 24 h, **K1** was added at final concentrations of 1–50 μM and cells were allowed to grow for additional 72 h under label-free conditions. The electrical impedance was measured by the RTCA-integrated software of the xCELLigence system (ACEA Biosciences) as a dimensionless parameter termed CI.

### 3.6. Experimental Design for Flow Cytometry Analysis

Caco2 cells (3 × 10^5^) were seeded in Petri dishes and cultivated 24 h in a complete medium with 10% FBS. Cells were treated with **K1** (c = 10 µM) for 12, 24, 48 and 72 h prior to analysis. The apoptosis, caspase 3 activation and cell cycle parameters were analysed 24, 48 and 72 h after treatment. Changes in MMP and ROS production were also analysed after 12 h of incubation. To evaluate ROS-dependent/independent mechanisms of **K1** treatment we used Trolox, a water-soluble analogue of vitamin E, as antioxidant. Trolox (300 μM) was used 1 h before **K1** treatment and after as a co-treatment with **K1** for 12, 24, 48 and 72 h before ROS and MMP analysis.

### 3.7. Analysis of Cell Cycle

For flow cytometric analysis (FCM) of the cell cycle, floating and adherent cells were harvested together 24, 48 and 72 h after treatment, washed in cold PBS, fixed in cold 70% ethanol and kept at −20 °C overnight. Prior to analysis, cells were washed twice in PBS, resuspended in staining solution (final concentration 0.1% Triton X-100, 0.5 mg/mL ribonuclease A and 0.025 mg/mL propidium iodide-PI), incubated in the dark at room temperature for 30 min and analysed using a FACSCalibur flow cytometer (Becton Dickinson, San Jose, CA, USA). 

### 3.8. Annexin V-FITC Labelling

The plasma membrane changes characteristic of apoptosis were analysed by double staining with Annexin V-FITC and PI according to the manufacturer’s instructions. Adherent and floating cells (1 × 10^5^) were harvested together 24, 48 and 72 h after treatment and stained with Annexin V-FITC (BD Biosciences Pharmingen, San Diego, CA, USA) in binding buffer for 15 min, washed, stained with PI for 5 min and thereafter analysed using a BD FACSCalibur flow cytometer. Three populations of cells were observed: viable cells: Annexin V-FITC negative and PI negative; apoptotic cells: Annexin V-FITC positive and PI negative; late apoptotic/necrotic cells: Annexin V-FITC positive and PI positive or and Annexin V-FITC negative and PI positive.

### 3.9. Measurement of ROS

The intracellular production of ROS was detected with FCM analysis using dihydrorhodamine-123 (DHR-123, Fluka), which reacts with intracellular hydrogen peroxide. The cells treated with an appropriate agent were harvested, washed twice in PBS, and resuspended in PBS. DHR-123 was added at a final concentration of 0.2 μM. The samples were then incubated for 15 min in dark and after incubation samples were placed on ice. Fluorescence was detected with 530/30 (FL-1) optical filter. Forward and side scatters were used to gate the viable populations of cells.

### 3.10. Detection of MMP

The changes in MMP were analysed with FCM using tetramethylrhodamine ethyl ester per chlorate (TMRE, Molecular Probes, Eugene, OR, USA). The cells were washed with PBS, resuspended in 0.1 μM of TMRE in PBS, and incubated for 30 min at room temperature in the dark. The cells were then washed twice with PBS, resuspended in 500 μM of the total volume, and analysed (1 × 10^4^ cell per sample). Fluorescence was detected with 585/42 (FL-2) optical filter.

### 3.11. Detection of Active Caspase 3

The changes in caspase 3 activation were analysed with FCM using BD Pharmingen Active Caspase-3 PE MAb Apoptosis kit (BD Bioscience, San Diego, CA, USA). The cells were prepared according to manufactory condition and stained with PE conjugated antibody and incubated for 30 min at room temperature in the dark. The cells were then washed twice with PBS, resuspended in 500 μM of the total volume, and analysed (1 × 10^4^ cell per sample). Fluorescence was detected with 585/42 (FL-2) optical filter.

### 3.12. DNA Fragmentation Assay

The culture medium was removed from untreated (1 × 10^6^) and treated Caco2 cells (**K1** 10 μM for 24, 48 and 72 h and 10 μM of cisplatin as positive control) and centrifuged at 1300 rpm for 5 min to collect them. Cells were washed twice with PBS calcium and magnesium free. Then cells were lysed in a lysis buffer containing 10 mmol/L EDTA, 0.5% Triton X-100. Proteinase K (1 mg/mL) was added and cells were incubated at 37 °C for 1 h followed by 10 min incubation at 70 °C. RNase (200 µg/mL) was added and cells were incubated for another 1 h at 37 °C. Samples were transferred to 2% agarose gel and run with 40 V for 3 h. DNA fragments were visualized by a UV illuminator. 

### 3.13. DAPI Staining

Twenty four hours after treatment (**K1** 10 μM), Caco2 cells grown on cover slips were fixed with 2% paraformaldehyde for 20 min at 4 °C. After incubation, the cells were washed briefly with PBS and incubated at room temperature with SlowFade® Gold antifade reagent with 4',6-diaminidino-2-phenyl-indole, dihydrochloride (DAPI) (Invitrogen) for nuclear visualization. The slides were analysed using fluorescence microscope Leica DMI6000 B (Leica Microsystems, Inc., Bannockburn, IL, USA) and evaluated as percentages of cells with a fragmented nucleus from a minimum of 300 cells.

### 3.14. RNA Isolation and cDNA Synthesis

Total RNA was isolated from Caco2 cells using the TRI Reagent (Molecular Research Center, Inc., Cincinnati, OH, USA) according to the manufacturer’s instruction. Total RNA quality was verified on an agarose gel. Total RNA (0.5 µg) was reverse transcribed into cDNA by the RevertAid^TM^ H Minus First Strand cDNA synthesis kit (Fermentas GmbH, St. Leon-Rot, Germany) according to the manufacturer’s instruction, and used for quantitative real time PCR.

### 3.15. Quantitative Real-Time PCR

Quantitative real time PCR analysis was performed in Light Cycler (Roche, Mannheim, Germany) using iQTM SYBR Green Supermix (Bio-Rad Laboratories, Hercules, CA, USA) to verify the alterations of α-tubulin, α_1_-tubulin, β_5_-tubulin gene expression. The PCR program was initiated by 5 min at 95 °C before 40 thermal cycles, each of 30 s at 95 °C and 45 s at 55 °C. Data were analysed according to the comparative Ct method and were normalized by β-actin expression in each sample. Melting curves for each PCR reaction were generated to ensure the purity of the amplification product.

### 3.16. Western Blot Analysis

Caco2 cells were treated with compound **K1** (10 μM), Trolox (300 μM) and mutual combinations for 24, 48 and 72 h. Protein extracts were obtained using a lysis buffer containing 100 mM Tris (pH 7.4), 1% SDS and 10% glycerol in the presence of PIC (protease inhibitor cocktail), for 30 min on ice. After the insoluble materials were removed by centrifugation at 12,000 *g* for 10 min at 4 °C, total protein concentrations were quantified using the Pierce® BCA Protein Assay Kit (Thermo Fisher Scientific, Waltham, MA, USA). Twenty micrograms of total cellular proteins were separated on 10% SDS polyacrylamide gels and electrotransferred onto nitrocellulose membranes (Pall Corporation, Port Washington, NY, USA). Membranes were blocked in 5% skim milk in Tris-buffered saline (TBS) containing 0.1% Tween-20 for 1 h at room temperature to minimize non-specific binding and incubated with the primary antibodies overnight at 4 °C. Immunoblotting was carried out with α Tubulin (E-19), α1c Tubulin (MH-87), β Tubulin (H-235) and β-Actin (C4) Antibody (all from Santa Cruz Biotechnology, Inc. Dallas, Texas, USA). After incubation with primary antibodies, membranes were washed 1 × 5 min with TBS-Tween followed by an incubation of 1 h at room temperature with the corresponding horseradish peroxidase-conjugated secondary antibodies (anti-rabbit IgG-HRP, anti-mouse IgG-HRP, all from Sigma-Aldrich). After washing 4 × 10 min with TBS-Tween expression was detected by chemiluminescence emission using ECL (Thermo Fisher Scientific) and then the blots were exposed to x-ray films. 

### 3.17. Statistical Analysis

Results are expressed as mean ± SD. Statistical analyses of the data were performed using standard procedures, with one-way ANOVA followed by the Bonferroni multiple comparisons test. Differences were considered significant when *p* values were smaller than 0.05.

## 4. Conclusions

In summary, the results revealed that **K1** exhibited the highest levels of antiproliferative activity against Caco2 colon cancer cells and was selected for follow-up study. The cell cycle results demonstrated that **K1** induced time-dependent G_2_/M arrest simultaneously with an increase in cells with sub-G_1_ DNA content. Furthermore, the apoptosis data showed that **K1** induced cellular apoptosis as confirmed by annexin V/PI double staining, DNA fragmentation and chromatin condensation. Moreover, increased ROS generation and loss of MMP and caspase 3 activation supported these findings. Application of Trolox, a water-soluble analogue of vitamin E, diminished Ros production and loss of MMP and prevented cytotoxicity of **K1** in Caco2 cells. These results insinuate that **K1** may inhibit the growth of colon cancer cells by inducing apoptosis through ROS-mitochondrial pathway. Although many details of the effects of **K1** remain to be elucidated, the *in vitro* findings of the present study provide the basis for future *in vitro* and *in vivo* studies.
